# Effectiveness of red yeast rice on carotid atherosclerosis: A systematic review and meta-analysis

**DOI:** 10.3389/fphar.2022.937809

**Published:** 2022-09-02

**Authors:** Shuai Wang, Yue Chen, Rui Wang, Bailing Ma, Zhenzhen Wang, Guanguang Tang, Siyu Wang, Yi He, Liping Qu

**Affiliations:** ^1^ Department of Psychology, Chengdu Medical College, Chengdu, China; ^2^ National Engineering and Research Center for Natural Medicines, Department of Medicine, Chengdu, China; ^3^ Department of Neurology, Xinxiang First People’s Hospital, Affiliated People’s Hospital of Xinxiang Medical University, Xinxiang, China; ^4^ State Key Laboratory of Southwestern Chinese Medicine Resources, Chengdu University of Traditional Chinese Medicine, Chengdu, China

**Keywords:** red yeast rice (RYR), meta-analysis, systematic review, carotid atherosclerosis, efficacy

## Abstract

While several studies have demonstrated the preventive and therapeutic effects of red yeast rice (RYR), a traditional Chinese medicine, on carotid atherosclerosis through the reduction of low-density lipoprotein cholesterol (LDL-C) level and other risk factors, the evidence remains inconsistent. This study aimed to further evaluate the effects of RYR in carotid atherosclerosis. Several databases were searched for original trials of RYR for the treatment of carotid atherosclerosis that reported plaque indicators. Carotid plaque area (AREA), carotid plaque score (SCORE), and intima-media thickness (IMT) were set as the primary outcomes, while lipid profile and safety indicators were set as the secondary outcomes. Meta-analyses were performed on the randomized controlled trials (RCTs) using Comprehensive Meta-analysis software. Heterogeneity was evaluated using the *I* ^2^ index and *Q* statistic. Subgroup, sensitivity, and dose-effect analyses were conducted. Twenty RCTs with 2217 patients were included. Compared to the control group, AREA (*SMD* = −0.855, *95%CI*: −1.259 to −0.451, *p* < 0.001), IMT (*SMD* = −0.588, *95%CI*: −0.792 to −0.384, *p* < 0.001), SCORE (*SMD* = −0.708, *95%CI*: −1.135 to −0.282, *p* = 0.001), LDL-C (*SMD* = −0.938, *95%CI*: −1.375 to −0.502, *p* < 0.001), triglyceride (*SMD* = −0.766, *95%CI*: −0.980 to −0.551, *p* < 0.001), and total cholesterol (*SMD* = −0.858, *95%CI*: −1.254 to −0.462, *p* < 0.001) were significantly decreased and HDL-C (*SMD* = 0.389, *95%CI*: 0.044–0.733, *p* = 0.027) was significantly increased following RYR therapy. The indicators for safety were not significant and did not differ between the two groups (*p* > 0.050). Heterogeneities mainly existed for the treatment time or control group setting. Most results showed no changes in the sensitivity analysis. Dose-effect relationships were observed for all indicators except for TC and HDL-C. We concluded that RYR therapy showed considerable efficacy and an acceptable safety profile for the treatment of carotid atherosclerosis in the Chinese population.

## Introduction

Carotid atherosclerosis, a key cause of ischemic stroke, is related to a high risk of cardiovascular events (Naqvi and Lee, 2014; Brinjikji et al., 2017). Among all cases of ischemic stroke, approximately 18%–25% can be attributed to atherosclerotic thromboembolic disease. The symptoms of carotid atherosclerosis include increased carotid intima-media thickness (IMT), plaques, and arterial stenosis. Although carotid atherosclerosis is not directly fatal, the long-lasting abnormal structure and function of carotid pathology lead to lower patient quality of life and a higher risk of cardiovascular events. Epidemiological investigations have verified a prevalence of carotid plaques of approximately 21.15% (Song et al., 2020).

Dyslipidemia affects the progression of carotid atherosclerosis. Dyslipidemia is a disease characterized by increased levels of total cholesterol (TC), low-density lipoprotein cholesterol (LDL-C), and triglyceride (TG), as well as a decreased level of high-density lipoprotein cholesterol (HDL-C) (Siavash et al., 2014). The goal in the treatment of these patients is to achieve target levels of LDL-C, and blood pressure and to inhibit plaque progression by reducing and eliminating the risk factors above (Spence, 2020). Lowering the LDL-C level is also one approach for atherosclerosis therapy. Statins are the first drug of choice for reducing LDL-C levels (Mach et al., 2020). Intensive statin therapy, such as 10–20 mg doses of rosuvastatin, can delay or even reverse atherosclerosis progression in western populations (Nissen, 2005; Nissen et al., 2006; Crouse et al., 2007; Du et al., 2014). However, these high doses showed less safety in Asian populations (Group, 2013). Diet therapy or traditional Chinese medicine (TCM) are other methods for the treatment of dyslipidemia to prevent the progression of carotid atherosclerosis (Gylling et al., 2014; Hollænder et al., 2015; Mach et al., 2020; Mojgan et al., 2020).

Red yeast rice (RYR) is a fermented rice. Its bright reddish-purple color occurs due to cultivation with the mold *Monascus purpureus*. RYR is also used in Chinese herbology and TCM to regulate lipid profiles and prevent cardiovascular events (Lu et al., 2008; Li et al., 2014; Fogacci et al., 2019). Chemical analysis showed that lovastatin (monacolin K) contains the same substances as occur in RYR (Xiong et al., 2019). The TCM treatments made from RYR include Zhibitai capsules, Zhibituo tablets, and Xuezhikang capsules. The chemical constituents of Zhibitai are RYR extract plus *Crataegus pinnatifida* Bunge, *Atractylodes macrocephala* Koidz, *Alisma plantago-aquatica* Linn (Xu et al., 2018). High-performance liquid chromatography analysis has shown that the currently known chemical components of Zhibitai mainly include: Monacolin K (Supplementary Figure S1), Ursolic acid, Oleanolic acid, Atractylide, and Alisitol, among others (China Heart Federation et al., 2017). The results of randomized controlled trials (RCTs) including patients with moderate to very high cardiovascular risks showed that Zhibitai lowered lipids by approximately 35% with lower risks of adverse reactions (China Heart Federation et al., 2017). Furthermore, Zhibitai treatment decreased plasma triglyceride (TG) levels much faster than atorvastatin (Xu et al., 2010; Xu et al., 2018). Zhibituo and Xuezhikang is a unilateral RYR preparation containing Mevastatin, Monacolin K, Daidzein, etc. (Supplementary Figure S2) (Lu et al., 2008). These components decreased lipid levels and cardiovascular events for the coronary secondary prevention population (Li et al., 2010). Thus, considering its lipid-lowering advantages and good tolerance in Chinese patients, RYR has received clinical attention. Clinical guidelines have described RYR as a moderate lipid-lowering medicine with ensured safety (Mach et al., 2020; Banach et al., 2021).

Evidence indicates that RYR drugs such as Zhibitai and Xuezhikang could decrease carotid atherosclerosis to a certain degree with moderate and comprehensive lipid-lowering effects that are suitable for Chinese patients with dyslipidemia (Zhang et al., 2004; Peng et al., 2020). However, the effects of RYR on carotid atherosclerosis remain controversial. Thus, the present study performed meta-analyses to further explore the effects of RYR therapies for carotid atherosclerosis.

## Materials and methods

### Registration

This systematic review and meta-analysis were registered in PROSPERO (CRD42020200692).

### Search strategy and selection criteria

This study followed the Preferred Reporting Items for Systematic Reviews and Meta-Analysis (PRISMA) 2020 statement (Page et al., 2021). The PubMed, Embase, ScienceDirect, and the Cochran Library databases, as well as the China National Knowledge Infrastructure (CNKI) and WANFANG DATA, two Chinese authoritative databases comprising hundreds of millions of global high-quality academic resources, were searched for English and Chinese language studies published between January 2012 and March 2022. The “PICOS” of the meta-analysis was: 1) patient (P): patients with carotid atherosclerosis; 2) intervention (I): treatment with RYR alone or in combination with other lipid-lower drugs; 3) control (C): treatment with statins, blank control, or placebo control; 4) outcomes (O): indicators of carotid plaques; 5) study design (S): RCTs. The search terms included the English and Chinese words “red yeast rice” or “Zhibitai” or “Zhibituo” or “Xuezhikang” in combination with “carotid/atherosclerosis/plaque” in these English or Chinese databases, respectively. The studies included in this review met the following inclusion criteria: 1) a prospective clinical trial with parallel-arm design; and 2) evaluation of the effect of RYR compared to placebo or control group on serum/plasma levels of lipid in carotid atherosclerosis. Studies were excluded if they met any of the following criteria: 1) RYR combined with non-lipid-lowering drugs for plaque treatment; 2) unavailable or missing data; 3) conference papers; and 4) duplicate publications. Two researchers independently performed the study search and selection. Language or publication was unlimited in the search process. Furthermore, additional potential eligible studies were identified by searching the reference lists of the identified studies.

### Data extraction and quality assessment

Two independent reviewers extracted the data. In the first screening, citation management software (EndNoteX8, Thomson Corporation) was used to carefully review the titles and abstracts of the eligible studies. Duplicates were removed. The full texts of the remaining studies were next evaluated to determine their inclusion in the analyses. Disagreements were resolved through discussion between the reviewers. Two reviewers independently extracted the specific information in the included articles, which was recorded in a pre-determined standard electronic form. The extracted contents were not limited to the following: 1) general study information (last name of the first author, publication year, and study type); 2) sample characteristics (number and age of patients in the RYR and control groups), 3) interventions; 4) follow-up duration; and 5) outcomes including carotid plaque area (AREA, the largest cross-sectional area of longitudinal views of all plaques measured by ultrasound), carotid plaque score (SCORE, mainly Crouse’s method calculated based on the number of ultrasound-detected plaques in the common, bifurcation, and internal carotid artery segments), IMT (the vertical dimension between carotid intima and media measured by ultrasound), TC, TG, LDL-C, HDL-C, creatinine (Cr), alanine aminotransferase (ALT), uric acid (UA), creatine kinase (CK), and aspartate aminotransferase (AST). Based on the results of large-scale clinical studies and guidelines, this systematic review defined therapeutic effectiveness or minimal clinically important differences (MCIDs) for AREA, SCORE, IMT, TC, TG, and LDL-C as significant decreases by 32%, 8%, 11%, 19%, 13%, and 31%, respectively, due to the clinical associations with the significant reduction of cardiovascular events (Wells et al., 2001; Group, 2002; Jellinger et al., 2017; Sun et al., 2020). The primary outcomes were effectiveness indicators for carotid atherosclerosis, including AREA, SCORE, and IMT. The secondary outcomes were the effectiveness indicators of the lipid profile (TC, TG, LDL-C, HDL-C) and safety indicators for adverse effects on liver or kidney functions caused by drug use, including Cr, ALT, AST, CK, and UA. Studies in which incomplete data could not be resolved by obtaining data from the original authors were excluded. The quality of the included studies was assessed by two independent reviewers according to the Cochrane Handbook for Systematic Reviews of Interventions (Cumpston et al., 2019). Quality assessment of the RCTs was assessed based on the revised Cochrane risk-of-bias tool for randomized trials (RoB 2) (Sterne et al., 2019). In this tool, the study quality was have: 1) a low risk of bias, if a low risk of bias was observed in all domains; 2) a moderate risk of bias, if some concerns were present in at least one domain; or 3) a high risk of bias, if a high risk of bias was observed in at least one domain or some concerns were present for multiple domains such that the confidence in the result was substantially decreased (Sterne et al., 2019; Barcot et al., 2021).

### Statistical analysis

Meta-analyses were conducted using Comprehensive Meta-analysis software version 3.0 (Wang et al., 2019). The systematic review was performed in accordance with Preferred Reporting Items for Systematic Reviews and Meta-Analyses (PRISMA) 2020 guidelines (Page et al., 2021). The effect size to which the treatment improved the indicators of carotid, lipid profile, and safety was defined as the mean ± standard deviation. The lipid profiles of all articles were reported as mmol/L. The standardized mean differences (SMDs) with 95% confidence interval (95%CIs) were recalculated. If studies differed in treatment time, RYR dose, or control group setting, a random-effects model was chosen. Heterogeneity among individual studies was evaluated using the *I* ^2^ index and *Q* statistic (Higgins et al., 2021). Heterogeneity was defined as follows: 1) 0%–40%, negligible; 2) 30%–60%, moderate heterogeneity; 3) 50%–90%, substantial heterogeneity; and 4) 75%–100%, considerable heterogeneity (Higgins et al., 2021). To investigate the influence of each study on the overall effect size, sensitivity analysis was conducted using the leave-one-out method (Zarezadeh et al., 2020). The sources of heterogeneity were further explored through subgroup analysis. RYR type (Zhibitai or Xuezhikang), treatment time (3, 6, or 12 months), dose (normal: 240 mg of Zhibitai twice daily or 600 mg of Xuezhikang twice daily; high: 480 mg of Zhibitai twice daily or 1200 mg of Xuezhikang twice daily), combination drugs (no, statin, or other), and different treatments in the control group (blank or statin) were set to categorize the cutoff points in subgroup analyses due to their potential as factors influencing the treatment efficacy and clinical medication and study design. The dose-effect relationships were explored by meta-regression (Jakubovski et al., 2019). Doi plots were then obtained based on the LFK index to evaluate the potential for publication bias in MetaXL (www.epigear.com) (Furuya-Kanamori et al., 2020). *P-*values *<*0.05 indicated significant differences between groups.

## Results

### Study characteristics

**FIGURE 1:**
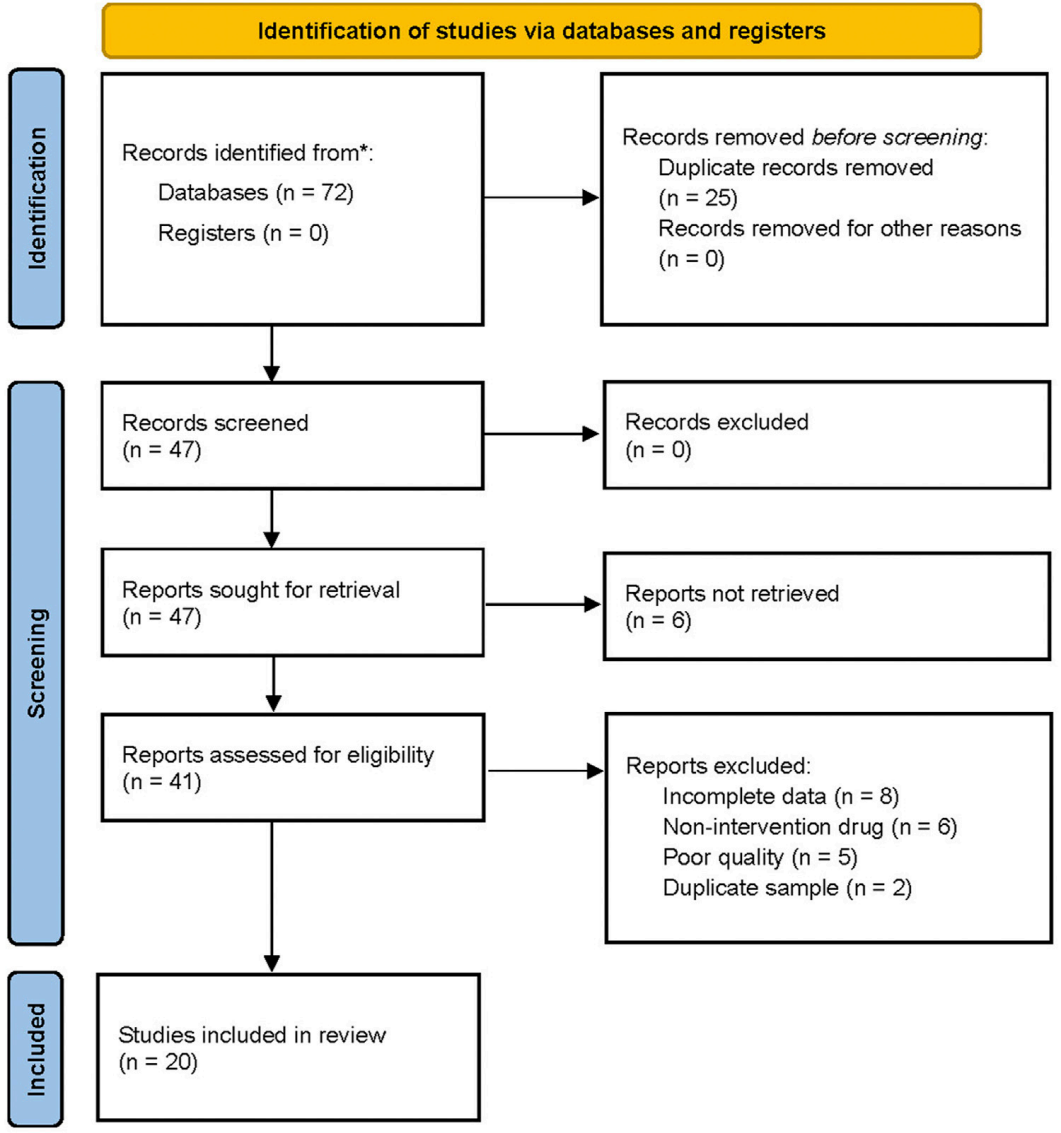
PRISMA flow diagram of study selection.

The flow chart for the search process is presented in Figure 1. The primary electronic search identified 72 potentially relevant studies. Twenty RCTs (Wu and Wang, 2012; Zhou et al., 2012; Zhu, 2012; Ao et al., 2013; Liu et al., 2013; Jiang and Chen, 2014; Liu, 2014; Yang et al., 2014; Tan, 2015; Zhang, 2015; Cui and Li, 2016; Pan et al., 2016; Su et al., 2016; Yang and Han, 2018; Jin, 2019; Li, 2020; Peng et al., 2020; Ma and Wang, 2021; Xia et al., 2021; Zhang, 2021) were included in the quantitative synthesis for the present systematic review and meta-analysis after screening and assessing the titles and abstracts for eligibility. The meta-analysis included a total of 2217 patients with carotid atherosclerosis, 422 of which in eight RCTs received Zhibitai-based therapy (Zhou et al., 2012; Yang et al., 2014; Tan, 2015; Yang and Han, 2018; Li, 2020; Peng et al., 2020; Ma and Wang, 2021; Xia et al., 2021), while 704 from 12 RCTs received Xuezhikang-based therapy (Wu and Wang, 2012; Zhu, 2012; Ao et al., 2013; Liu et al., 2013; Jiang and Chen, 2014; Liu, 2014; Zhang, 2015; Cui and Li, 2016; Pan et al., 2016; Su et al., 2016; Jin, 2019; Zhang, 2021). The details of the general characteristics and outcomes of the 20 studies are presented in Table 1. The details of isolated chemical compounds of the RYR used in these studies are presented in Supplementary Table S1. Two studies (Jiang and Chen, 2014; Zhang, 2021) had three arms, which constituted two sets of comparisons, respectively. Hence, the meta-analysis included a total of 22 sets of comparisons.

**TABLE 1:** General characteristics of the included studies.

Study and year	No. of Zhibitai group	Age of Zhibitai group, y	No. of control group	Age of control group, y	Interventions in RYR group	Interventions in control group	Follow-up, mo	Outcomes
Zhou et al. (2012)	69	59.1 ± 9.2	70	61.4 ± 8.5	Zhibitai 240 mg bid	Simvastatin 20 mg qd	12	AREA, IMT, TC, TG, LDL-C, HDL-C, Cr, UA, ALT, CK
Wu and Wang (2012)	18	51–73	13	51–73	Xuezhikang 600 mg tid	Blank	6	IMT, TC, TG
Zhu (2012)	30	63.4	35	60.5	Xuezhikang 600 mg bid	Simvastatin 20 mg qd	3	IMT, TC, TG, LDL-C, HDL-C
Ao et al. (2013)	60	64.5 ± 10.0	60	63.6 ± 10.2	Xuezhikang 600 mg bid + Atorvastatin 20 mg qd	Blank	3	IMT, TC, TG, HDL-C
Liu et al. (2013)	42	63.4 ± 10.5	40	62.9 ± 10.3	Xuezhikang 600 mg bid + Rosuvastatin 20 mg qd	Rosuvastatin 20 mg qd	12	AREA, IMT, TG, LDL- C
Jiang and Chen (2014)	30	NS	30 or 30	NS	Xuezhikang 600 mg bid	Blank or Rosuvastatin 10 mg qd	6	IMT, TC, TG, LDL-C, HDL-C
Yang et al. (2014)	30	68.6 ± 8.3	30	69.2 ± 7.5	Zhibitai 240 mg bid	Atorvastatin 10 mg qd	3	AREA, SCORE, IMT, TC, TG, LDL-C, ALT, AST
Liu (2014)	30	58.0 ± 5.4	30	58.0 ± 5.4	Xuezhikang 600 mg bid	Blank	6	IMT, AREA, LDL-C, TC, TG, HDL-C
Tan (2015)	30	67.9 ± 10.3	30	66.1 ± 11.3	Zhibitai 480 mg bid+ Simvastatin 10 mg qd	Simvastatin 20 mg qd	3	LDL-C, SCORE, ALT, IMT, TC, TG, HDL-C, Cr, CK, UA
Zhang (2015)	61	61.2 ± 12.0	61	60.8 ± 11.9	Xuezhikang 600 mg bid	Blank	2	IMT, TC, TG, LDL-C, HDL-C
Cui and Li (2016)	58	63.15 ± 9.18	58	62.85 ± 9.26	Xuezhikang 600 mg bid + Atorvastatin 20 mg qd	Atorvastatin 20 mg qd	12	AREA, SCORE, IMT, TC, TG, LDL-C
Pan et al. (2016)	152	50.1 ± 7.92	150	49.5 ± 7.36	Xuezhikang 600 mg bid	Blank	12	AREA, TC, TG, LDL-C, UA
Su et al. (2016)	53	61.1 ± 10.8	55	60.1 ± 10.2	Xuezhikang 600 mg bid	Blank	6	IMT, TC, TG, LDL-C, HDL-C
Yang and Han (2018)	30	70.3 ± 9.3	30	70.2 ± 10.2	Zhibitai 240 mg bid	Blank control	3	LDL-C, ALT, AREA, TG, SCORE, IMT, TC, HDL-C, AST
Jin (2019)	50	62.0 ± 6.7	46	61.2 ± 6.5	Xuezhikang 600 mg bid + Rosuvastatin 10 mg qd	Rosuvastatin 10 mg qd	3	AREA, IMT, TC, TG, LDL-C, HDL-C
Peng et al. (2020)	62	62.3 ± 7.9	62	61.6 ± 7.3	Zhibitai 480 mg bid	Atorvastatin 10 mg qd	6	LDL-C, AREA, IMT, TG, HDL-C, ALT
Li (2020)	36	60.5 ± 8.8	36	58.9 ± 9.5	Zhibitai 240 mg bid + Atorvastatin 10 mg qd	Atorvastatin 10 mg qd	3	IMT, SCORE, LDL-C, TC, TG, HDL-C
Xia et al. (2021)	90	67.9 ± 4.3	90	68.7 ± 3.7	Zhibitai 240 mg bid + Atorvastatin 20 mg qd	Atorvastatin 40 mg qd	6	IMT, SCORE, LDL-C, TC, TG, HDL-C
Ma and Wang (2021)	75	53.12 ± 5.67	75	52.68 ± 6.18	Zhibitai 480 mg bid	Atorvastatin 20 mg qd	3	IMT, SCORE, AREA, LDL-C, TC, TG, HDL-C, ALT, AST, CK
Zhang (2021)	60 or 60	64.40 ± 8.47 or 62.63 ± 10.17	60	63.45 ± 9.88	Xuezhikang 600 mg bid or Xuezhikang 600 mg bid + Rosuvastatin 5 mg qd	Rosuvastatin 10 mg qd	6	IMT, SCORE, TC, TG, LDL-C, HDL-C, Cr, UA, ALT, AST, CK

### Quality and publication bias assessments

**FIGURE 2:**
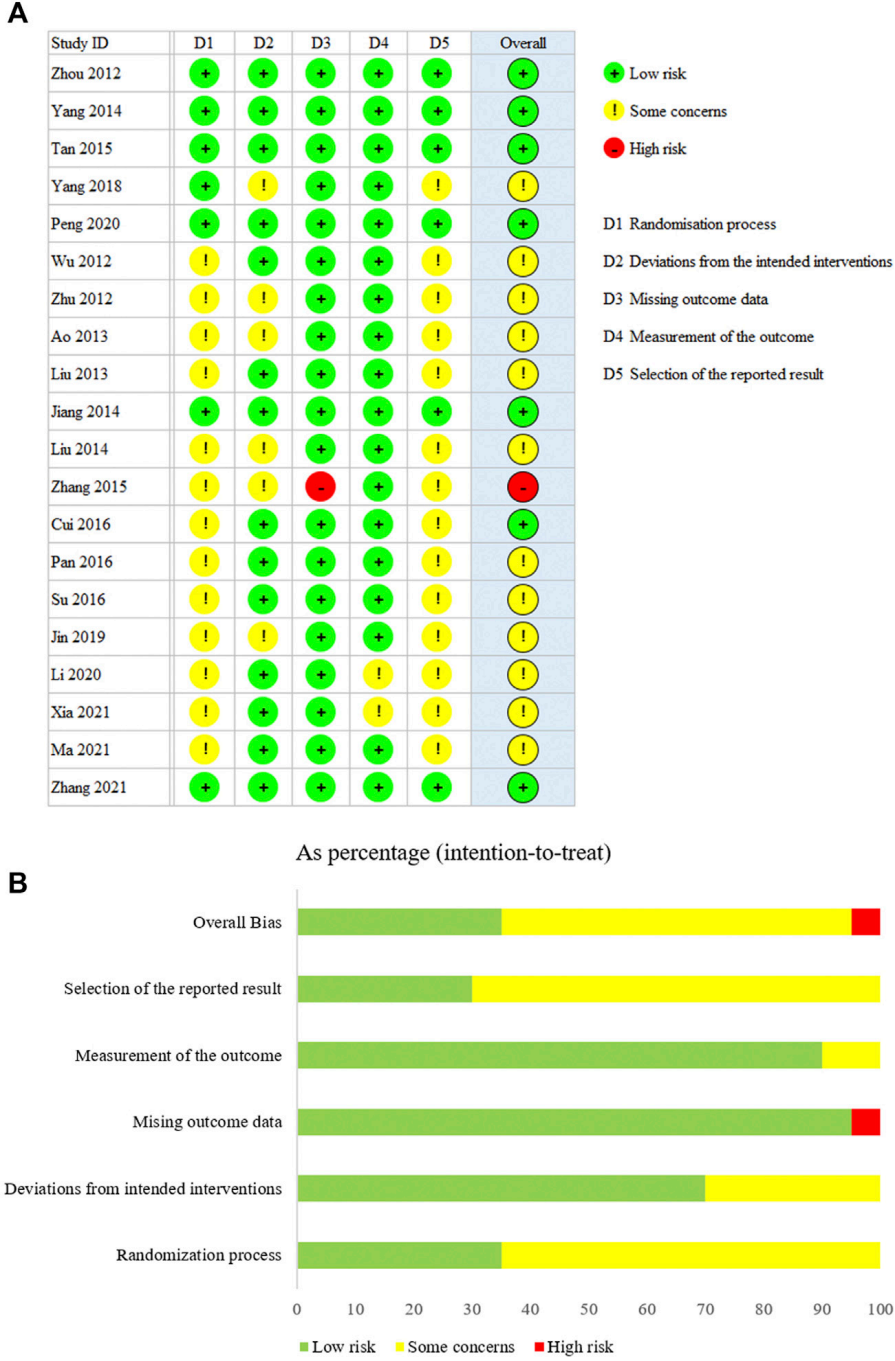
Evaluation of the risk of bias risk among the included studies. **(A)** Summary. **(B)** Overall.

The results of the assessments of the methodology qualities of the RCTs are shown in Figure 2. Overall bias was judged to show “some concerns” regarding bias for 12 of the trials. One trial was judged as having a “high risk”. Seven trials were judged to be at “low risk” for all five dimensions. In addition, according to the LFK index test, potential major publication bias was found only for Cr (Table 2). The others showed no or minor asymmetries.

**TABLE 2:** Publication bias of the meta-analysis.

Outcomes	Number of studies (comparators)	LFK index (Doi plot)
AREA	10 (10)	−1.97 (Minor asymmetry)
IMT	18 (20)	0.03 (No asymmetry)
SCORE	10 (12)	−0.77 (No asymmetry)
LDL-C	17 (19)	−0.72 (No asymmetry)
TC	18 (20)	−1.58 (Minor asymmetry)
TG	20 (22)	−1.03 (Minor asymmetry)
HDL-C	14 (16)	0.81 (No asymmetry)
ALT	6 (6)	0.91 (No asymmetry)
AST	2 (2)	—
CK	3 (3)	−1.56 (Minor asymmetry)
Cr	3 (3)	−2.38 (Major asymmetry)
UA	3 (3)	−0.39 (No asymmetry)

### Effectiveness of red yeast rice therapy on carotid atherosclerosis

**FIGURE 3:**
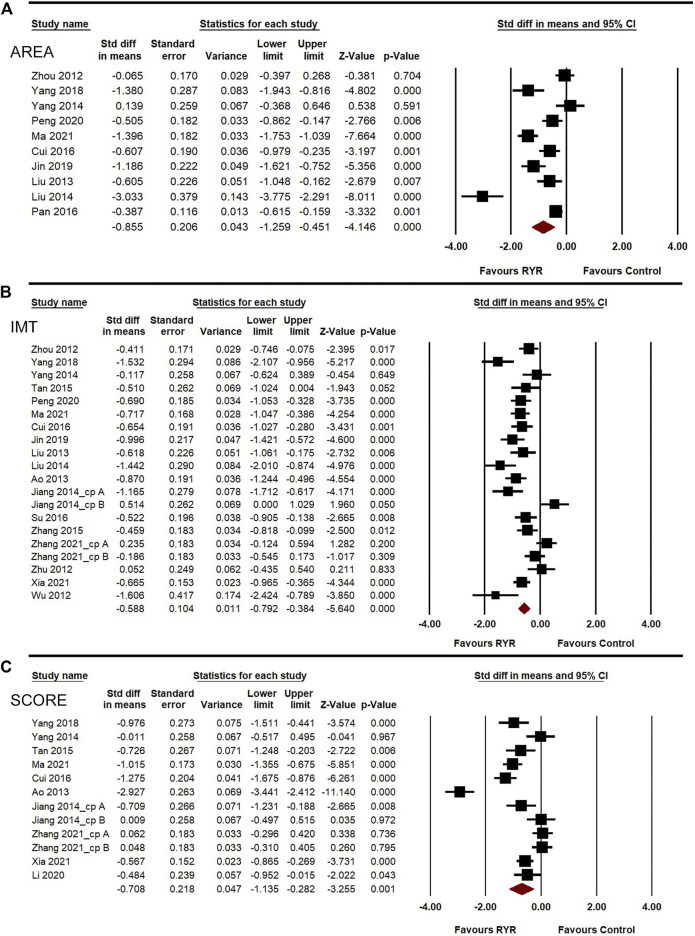
Meta-analysis of the effects of RYR therapy on AREA **(A)**, IMT **(B)**, and SCORE **(C)**.

AREA, IMT, and SCORE were used to assess the effects of RYR on carotid atherosclerosis. The heterogeneities of AREA, IMT, and SCORE among the studies were high (*I* ^*2*^ = 90.601%, 78.853%, and 91.772%, respectively). A random-effects model was used for the meta-analysis of these indicators. Compared to the control group, RYR therapy was associated with significant reductions in AREA (SMD = –0.855, 95%CI: −1.259 to −0.451, *Z* = −4.146, *p* < 0.001; Figure 3A), IMT (SMD = −0.588, 95%CI: −0.792 to −0.384, *Z* = −5.64, *p* < 0.001; Figure 3B), and SCORE (SMD = −0.708, 95%CI: −1.135 to −0.282, *Z* = −3.255, *p* = 0.001; Figure 3C). In the sensitivity analysis, no single study remarkably affected the effect sizes of AREA, IMT, and SCORE.

### Effectiveness of red yeast rice therapy on lipid lowering

**FIGURE 4:**
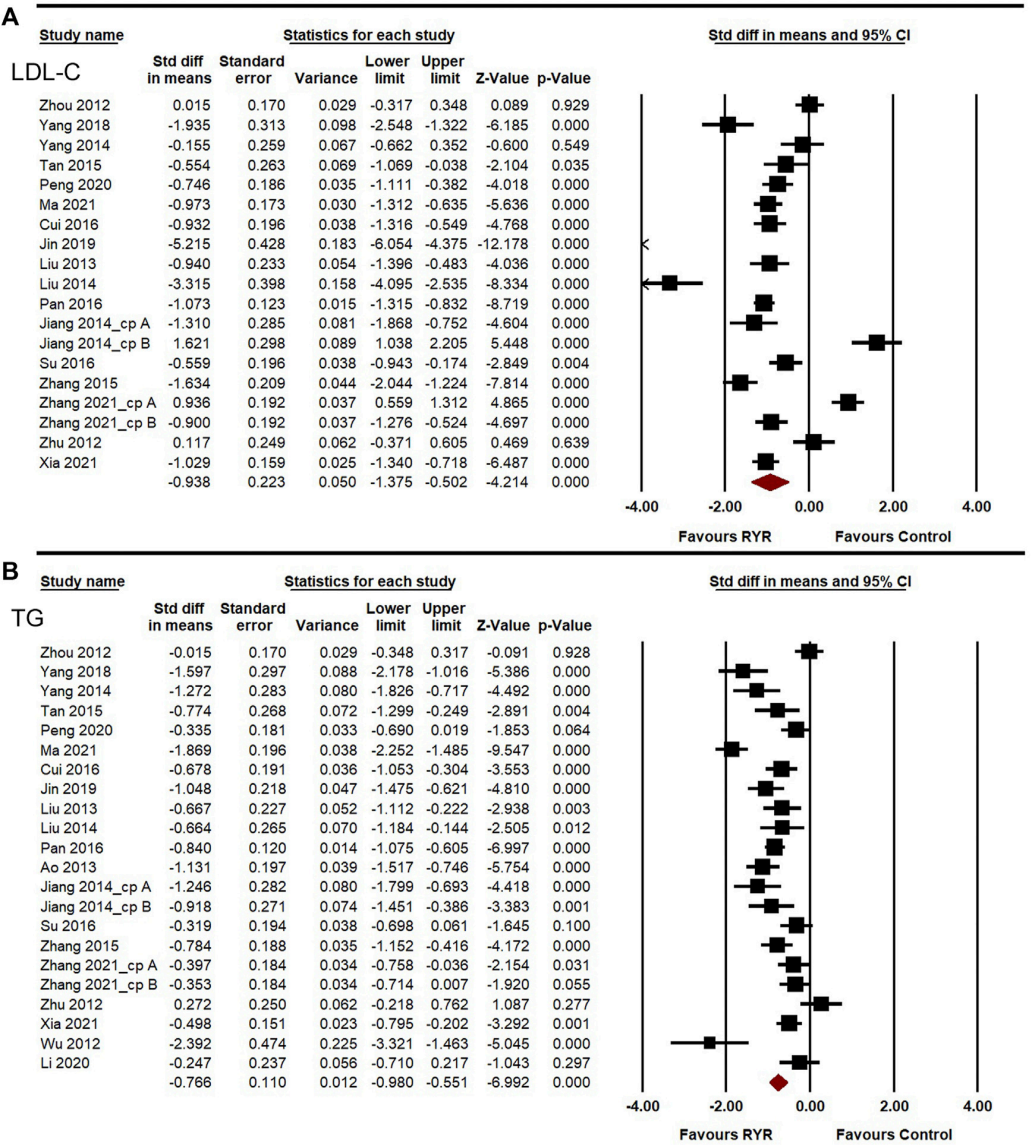
Meta-analysis of the effects of RYR therapy on LDL-C **(A)** and TG **(B)**.

**FIGURE 5:**
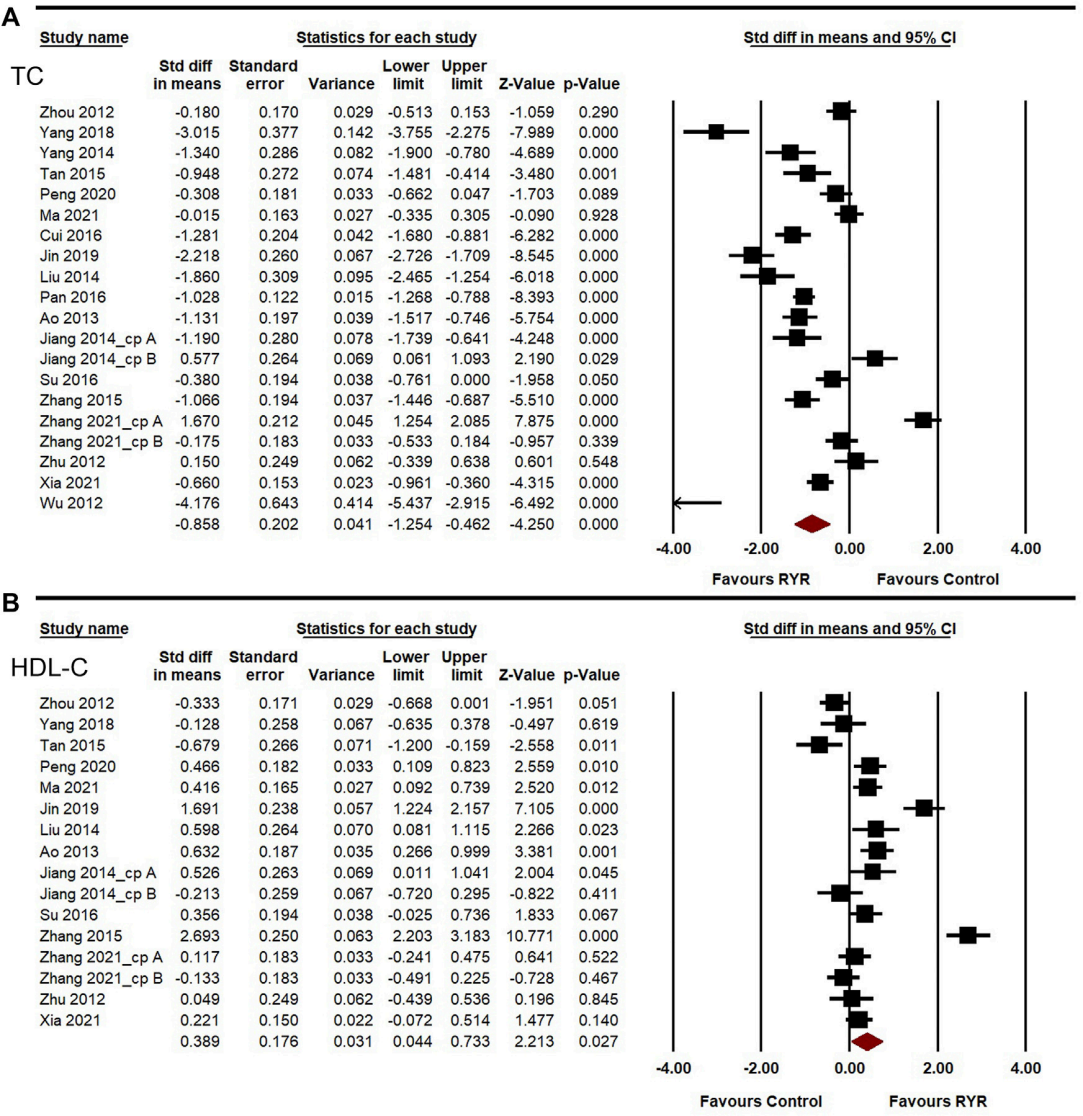
Meta-analysis of the effects of RYR therapy on TC **(A)** and HDL-C **(B)**.

Lipid-lowering ability, especially that for LDL-C and TG, is also a crucial aspect of assessing the effectiveness of therapies against carotid atherosclerosis. The heterogeneities of LDL-C, TG, TC, and HDL-C among the studies were high (*I* ^*2*^ = 95.260%, 83.346%, 94.542%, and 91.365%, respectively). A random-effects model was used for the meta-analysis of these indicators. Compared to the control group, RYR therapy was associated with significant reductions in LDL-C level (SMD = −0.938, 95%CI: −1.375 to −0.502, *Z* = −4.214, *p* < 0.001; Figure 4A), TG level (SMD = −0.766, 95%CI: −0.980 to −0.551, *Z* = −6.992, *p* < 0.001; Figure 4B), TC level (SMD = −0.858, 95%CI: −1.254 to −0.462, *Z* = −4.250, *p* < 0.001; Figure 5A), and HDL-C increment (SMD = 0.389, 95%CI: 0.044 to 0.733, *Z* = 2.213, *p* = 0.027; Figure 5B). In the sensitivity analysis, no single study remarkably affected the effect sizes of LDL-C, TG, and TC. However, after removing the studies by Jin 2019 (Jin, 2019) or Zhang 2015 (Zhang, 2015), the result for HDL-C was no longer significant. (SMD = 0.303, 95%CI: −0.025 to 0.631, *Z* = 1.812, *p* = 0.070; SMD = 0.240, 95%CI: −0.010 to 0.491, *Z* = 1.885, *p* = 0.059).

### Safety of red yeast rice therapy on carotid atherosclerosis

The effects of RYR therapy on safety are shown in Table 3. The heterogeneity of ALT, AST, and UA among studies were high (*I* ^*2*^ = 88.945%, 98.669%, and 76.336%, respectively); however, the heterogeneity of CK and Cr was low (*I* ^*2*^ = 24.664% and 16.811%, respectively). No significant differences were observed in ALT, AST, CK, and UA levels. Compared to the control group, the SMD of RYR therapy on Cr was −0.233 (*95%CI*: −0.452 to−0.014, *Z* = −2.084, *p* = 0.037). In the sensitivity analysis, no single study remarkably affected the effect size of ALT, AST, CK, and UA. However, after removing the studies by Zhou et al., 2012 (Zhou et al., 2012) or Tan 2015 (Tan, 2015), the result for Cr was no longer significant. (*SMD* = −0.276, *95%CI*: −0.567 to 0.015, *Z* = −1.857, *p* = 0.063; *SMD* = −0.153, *95%CI:* −0.395 to 0.089, *Z* = −1.237, *p* = 0.216).

**TABLE 3:** Meta-analysis of the safety of RYR therapy on the carotid atherosclerosis.

Outcomes	No. of studies	*I* ^2^ (%)	Effect model	SMD	*95%CI*	*Z* value	*p* value
ALT	6	88.945	Random	0.314	−0.197 to 0.825	1.201	0.229
AST	2	98.669	Random	−0.995	−3.883 to 1.892	−0.675	0.499
CK	3	24.664	Fixed	−0.157	−0.367 to 0.054	−1.461	0.144
Cr	3	16.811	Fixed	−0.233	−0.452 to −0.014	−2.084	0.037
UA	3	76.336	Random	−0.072	−0.551 to 0.406	−0.296	0.767

### Subgroup analysis of red yeast rice therapy on carotid atherosclerosis and lipid lowering

Subgroup analyses were conducted to explore the source of heterogeneity and to further determine RYR effectiveness. When the studies were stratified by RYR type, Zhibitai (*SMD* = −0.641, *Z* = −4.480, *p* < 0.001) but not Xuezhikang (*SMD* = −0.792, *Z* = −1.797, *p* = 0.072) therapy was associated with a significant reduction in SCORE. Xuezhikang therapy was associated with a significant increment in HDL-C (*SMD* = 0.627, *Z* = 2.362, *p* = 0.018) (Table 4). When the studies were stratified by treatment time, 12 months of RYR therapy was more effective on AREA, IMT, and SCORE (Table 5). The effects of RYR therapy on carotid plaques and lipid profiles did not change significantly when the studies were stratified by RYR dose, combination drugs, or control setting, except for SCORE (combination drugs), TC (control setting), and HDL-C (RYR dose, combination drugs, and control setting) (Tables 6–8). The heterogeneities were significantly decreased or no longer present in the subgroup analyses.

**TABLE 4:** Subgroup analysis stratified by RYR type.

Outcomes	Zhibitai			Xuezhikang		
	SMD	*Z* value	*p* value	SMD	*Z* value	*p* value
AREA	−0.636	−2.050	0.040	−1.090	−3.397	0.001
IMT	−0.642	−5.183	<0.001	−0.561	−3.706	<0.001
SCORE	−0.641	−4.480	<0.001	−0.792	−1.797	0.072
LDL-C	−0.749	−3.539	<0.001	−1.062	−3.049	0.002
TG	−0.810	−3.343	0.001	−0.734	−6.515	<0.001
TC	−0.855	−3.214	0.001	−0.863	−2.989	0.003
HDL-C	0.022	0.130	0.896	0.627	2.362	0.018

**TABLE 5:** Subgroup analysis stratified by treatment time.

Outcomes	Three months			Six months			Twelve months		
	SMD	*Z* value	*p* value	SMD	*Z* value	*p* value	SMD	*Z* value	*p* value
AREA	−0.963	−2.786	0.005	−1.747	−1.382	0.167	−0.394	−3.321	0.001
IMT	−0.639	−4.197	<0.001	−0.571	−2.887	0.004	−0.543	−4.891	<0.001
SCORE	−1.021	−2.704	0.007	−0.225	−1.346	0.178	−1.275	−6.261	<0.001
LDL-C	−1.437	−3.056	0.002	−0.641	−1.646	0.100	−0.731	−2.713	0.007
TG	−0.939	−4.353	<0.001	−0.661	−4.821	<0.001	−0.552	−2.776	0.006
TC	−1.170	−3.594	<0.001	−0.614	−1.756	0.079	−0.827	−2.642	<0.001
HDL-C	0.669	1.715	0.086	0.231	2.407	0.016	−0.333	−1.951	0.051

**TABLE 6:** Subgroup analysis stratified by dosage of RYR.

Outcomes	Normal			High		
	SMD	*Z* value	*p* value	SMD	*Z* value	*p* value
AREA	−0.834	−3.444	0.001	−0.950	−2.133	0.033
IMT	−0.538	−4.380	<0.001	−0.761	−4.990	<0.001
SCORE	−0.676	−2.597	0.009	−0.929	−6.389	<0.001
LDL-C	−0.980	−3.650	<0.001	−0.809	−7.098	<0.001
TG	−0.667	−6.730	<0.001	−1.295	−2.796	0.005
TC	−0.801	−3.466	0.001	−1.114	−2.377	0.017
HDL-C	0.460	2.181	0.029	0.100	0.321	0.748

**TABLE 7:** Subgroup analysis stratified by combination drugs.

Outcomes	No combination			Statin		
	SMD	*Z* value	*p* value	SMD	*Z* value	*p* value
AREA	−0.895	−3.112	0.002	−0.793	−4.184	<0.001
IMT	−0.568	−3.614	<0.001	−0.640	−6.411	<0.001
SCORE	−0.440	−1.953	0.051	−0.978	−2.605	0.009
LDL-C	−0.674	−2.490	0.013	−1.518	−3.779	<0.001
TG	−0.837	−5.045	<0.001	−0.669	−6.108	<0.001
TC	−0.783	−2.918	0.004	−1.052	−4.052	<0.001
HDL-C	0.408	1.858	0.063	0.348	1.048	0.295

**TABLE 8:** Subgroup analysis stratified by control setting.

Outcomes	Blank			Statin		
	SMD	*Z* value	*p* value	SMD	*Z* value	*p* value
AREA	−1.564	−2.145	0.032	−0.609	−2.956	0.003
IMT	−1.017	−5.770	<0.001	−0.382	−3.368	0.001
SCORE	−1.538	−2.179	0.029	−0.446	−2.610	0.009
LDL-C	−1.571	−5.435	<0.001	−0.640	−2.265	0.024
TG	−1.015	−6.387	<0.001	−0.621	−4.375	<0.001
TC	−1.550	−6.029	<0.001	−0.387	−1.511	0.131
HDL-C	0.778	2.093	0.036	0.163	0.945	0.345

### Dose-effect relationship

The results of the meta-regression analyses reflected a significant negative relationship between RYR dose and SMD in AREA, IMT, SCORE, LDL-C, and TG (All *p* < 0.05, Table 9). However, no obvious and meaningful relationships between RYR dose and SMD were observed for TC or HDL-C.

**TABLE 9:** Effect of dosage on measured efficacy of RYR.

Outcomes	*Β* value	*95%CI*	*Z* value	*p* value
AREA	−0.484	−0.785 to −0.183	−3.150	0.002
IMT	−0.394	−0.666 to −0.122	−2.843	0.004
SCORE	−0.772	−1.171 to −0.373	−3.789	<0.001
LDL-C	−0.320	−0.597 to −0.042	−2.260	0.024
TG	−0.513	−0.791 to −0.235	−3.620	<0.001
TC	0.267	0.003 to 0.531	1.979	0.048
HDL-C	0.127	−0.153 to 0.408	0.890	0.373

## Discussion

This systematic review and meta-analysis explored the efficacy and safety of RYR on the indicators of carotid atherosclerosis as well as lipid profiles. Although limited articles were included and deemed eligible for this systematic review, most studies had low risk and demonstrated significant improvements in the indicators of carotid atherosclerosis and various plasma/serum lipid biomarkers. The present meta-analysis showed that carotid atherosclerosis and lipid but not safety outcomes differed significantly between the RYR and control groups, although the Cr levels were lower in the RYR group. All studies were clinical trials, among which two studies had three-arm designs; the other 18 studies had double-arm designs, which increased the power of the research. However, due to the influence of various factors, including the number of participants, RYR type, treatment time, RYR dose, and variable inconsistency, this study had relatively high heterogeneity and it was difficult to draw clear conclusions.

Carotid atherosclerosis is an atherosclerosis cardiovascular disease (ASCVD) closely related to the occurrence of ischemic stroke and cardiovascular diseases (Gupta et al., 2013; Kawai et al., 2013; Park et al., 2013). ASCVD events can be reduced through effective treatment of carotid atherosclerosis, which has significant clinical benefits for patients (CD et al., 1994; Zhang et al., 2019). The indicators of carotid atherosclerosis may also be independent risk markers for cardiovascular and cerebrovascular diseases, which could predict the occurrence, development, and prognosis of cardiovascular and cerebrovascular events (Spence et al., 2002; Spence and Hackam, 2010; Mathiesen. E et al., 2011; Park et al., 2013). Further analysis of the included RCTs in the present study demonstrated the potential advantage of RYR treatment by improving AREA, IMT, and SCORE compared to those in the control group (including statin treatment), although the result for SCORE was unstable in subgroup analyses. Zhibitai may have more advantages in improving SCORE. Among the included studies with 6 months’ treatment duration, those by Su et al., 2016 (Su et al., 2016) and Zhang 2021 (Zhang, 2021) showed negative results for some indicators with relatively high weight for the comparison of Xuezhikang to statins. Thus, the results of the subgroup analysis were not significant. Furthermore, dose-effect relationships exist between RYR and carotid atherosclerosis. The results of the systematic review and meta-analysis of the main indicators of carotid atherosclerosis in the present study suggested the potentially significant value of RYR for the reduction of ASCVD events by delaying the progression of carotid atherosclerosis.

Dyslipidemia tends to be the most crucial risk factor associated with atherosclerosis. Therefore, lipid regulation is particularly critical for preventing atherosclerosis progression (Virani et al., 2012; Mi et al., 2016). The patients in all 20 studies had dyslipidemia. Statistical analysis indicated that RYR effectively improved LDL-C, TG, TC, and HDL-C levels (all *p* < 0.05). RYR had a moderate and comprehensive lipid-lowering ability. Furthermore, the results of the dose-effect analysis suggested that a higher dosage of RYR had a higher efficacy on improving these indicators. The results of the TWINS and COSMOS studies, which used low-dosage statin as an intervention, suggested that moderate lipid-lowering therapy can reduce plaque size in Japanese patients (Hiro et al., 2009; Takayama et al., 2009). The results of the present study showed that long-term treatment with a moderate lipid-lowering drug (RYR) can reverse plaques in Asian patients, especially in Chinese populations. Therefore, the use of RYR has increased the drug choices for the Chinese population.

The results of this study also demonstrated the good safety of both RYR and the control groups. The results of the safety analysis showed no abnormal changes in these safety measures before and after treatment compared to those in the control group. Although Cr levels were lower in the RYR group, this difference was no longer significant in the sensitivity analysis. These results suggested that there was no need for dosage adjustment and replacement drugs during treatment with RYR, and that only monitoring of liver function was needed. Proteinuria and impaired kidney function with the use of these medications is another concern. Notably, RYR also showed an additional Cr-lowering effect. The results may help doctors to choose suitable therapy for individual patients based on the clinical efficacy of RYR on carotid atherosclerosis, as well as its economic cost and safety factors.

This meta-analysis had several obvious heterogeneities for which subgroup analyses were performed to detect the sources. The heterogeneities were significantly decreased in most outcomes. The treatment time and the control group setting contributed most to the heterogeneities. In addition, the clinical characteristics of the participants, such as carotid atherosclerosis stage, differences in body mass index, lifestyle, comorbidities, carotid plaque measurements, and use of other drugs were factors potentially influencing the treatment outcomes (Irace et al., 2009; Jhamnani et al., 2015; Akhtar et al., 2018). However, the studies included in the meta-analysis contained all stages of carotid atherosclerosis, body-mass index, lifestyle, etc., which could not be stratified by these different baselines. These may have contributed to the heterogeneities in the primary and secondary outcomes. Overall, this analysis systematically searched for studies assessing the effectiveness of RYR on carotid atherosclerosis among Chinese patients. The synthesis and analysis of scattered, independent, and small sample-size studies provided a reliable conclusion to further confirm the effectiveness of RYR therapy for carotid atherosclerosis.

This study has several strengths. First, this study comprehensively analyzed the efficacy of RYR in RCTs, which increased the reliability of the results. Second, not only carotid plaque but also lipid levels and safety indicators were investigated, which adds to the evidence regarding the significance of TCM for the treatment of ASCVD. This study also has several limitations: 1) a significant publication bias was observed for Cr, which may affect the interpretation of the results; 2) the numbers of included RCTs and sample sizes were relatively small; 3) although the heterogeneity was decreased in subgroup analyses, it was still present in some outcomes, which might impair the strength of the conclusions; and 4) the observational outcomes were mostly soft endpoints and alternative indicators and the follow-up duration was short; 5) most included studies published in Chinese journals only passed the ethics committee and were not registered in Clinicaltrilas.gov, possibly due to insufficient awareness of registration among Chinese scholars in the past decade (Wu et al., 2018). A series of registered, well-designed large-scale, multi-center, placebo-parallel, and sufficiently followed-up RCTs are needed to further refine the evaluation.

## Conclusion

The results of the present systematic review and meta-analysis demonstrated the considerable efficacy and safety profile of RYR therapy presented for the treatment of carotid atherosclerosis in Chinese patients. Thus, RYR is a safe and effective traditional Chinese medicine that can be considered for the treatment of carotid plaques. However, large-scale and multicenter RCTs on this topic are also needed due to the current research status.

## Data Availability

The original contributions presented in the study are included in the article/Supplementary Material, and further inquiries can be directed to the corresponding authors.
