# Supplementary material for: Effectiveness of red yeast rice on carotid atherosclerosis: A systematic review and meta-analysis
Source: Front Pharmacol. 2022 Sep 2;13:937809. doi: 10.3389/fphar.2022.937809 (PMC9478999; doi:10.3389/fphar.2022.937809)
Supplement: Supplementary file 1 [file DataSheet1.docx]

**SUPPLEMENTARY MATERIALS**

**SUPPLEMENTAL FIGURE 1** High performance liquid chromatography (HPLC) of RYR in Zhibitai capsule.


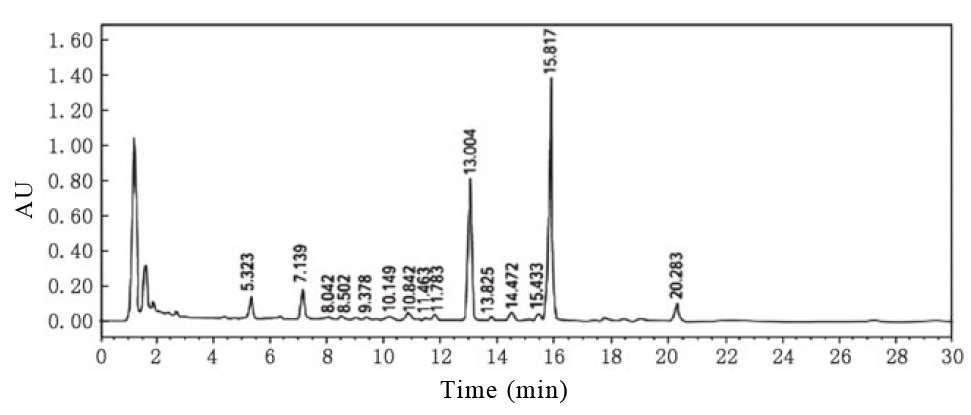


13.004, Closed-loop Monacolin K; 15.817, Open-loop Monacolin K

*Federation, C.H., Rehabilitation, C.S.o.C.P.a., and Capsules, T.W.G.o.C.E.C.o.t.U.o.Z. (2017). Chinese expert consensus on the use of Zhibitai capsules. Chin Int Med 56(8), 628-632. doi: 10.3760/cma.j.issn.0578-1426.2017.08.016.*

**SUPPLEMENTAL FIGURE 2** High performance liquid chromatography (HPLC) of RYR in Xuezhikang capsule.


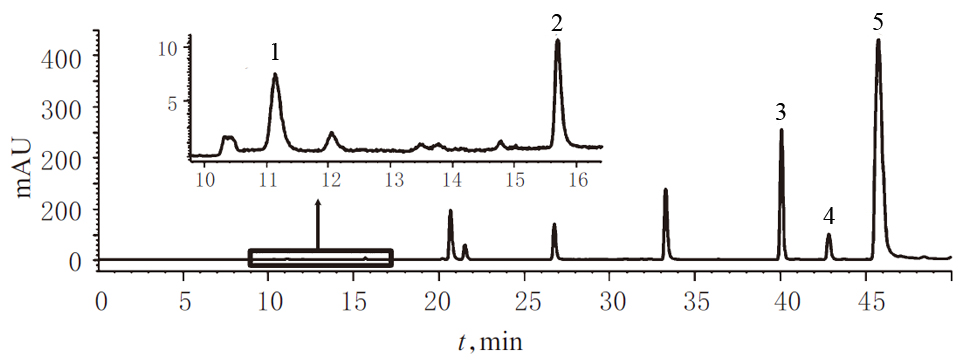


1, Daidzein; 2, Genistein; 3, Monacolin K hydroxy acid; 4, Mevastatin; 5, Monacolin K

*Lu, Z., Kou, W., Du, B., Wu, Y.F., Zhao, S., Brusco, O., et al. (2008). Effect of Xuezhikang, an extract from red yeast chinese rice, on coronary events in a chinese population with previous myocardial infarction. Am J Cardiol 101(12), 1689-1693. doi: 10.1016/j.amjcard.2008.02.056.*

*Li, Y., Sun X. (2019). Simultaneous content determination of 8 components in Xuezhikang capsules by HPLC-DAD. China Pharmacy 30(15), 2066-2070. Doi: 10.6039/j.issn.1001-0408.2019.15.11.*

**SUPPLEMENTAL TABLE 1** Isolated chemical compound in RYR.

| Study | Compound, concentration | Source | Purity (%) | Quality control  reported? (Y/N) |
| --- | --- | --- | --- | --- |
| Zhou 2012 | Monacolin K, 5.0 mg  Monacolin complex | Commercial supplier | Not applicable | Y-HPLC |
| Wu 2012 | Monacolin K, 2.5 mg  Monacolin complex | Commercial supplier | Not applicable | Y-HPLC |
| Zhu 2012 | Monacolin K, 2.5 mg  Monacolin complex | Commercial supplier | Not applicable | Y-HPLC |
| Ao 2013 | Monacolin K, 2.5 mg  Monacolin complex | Commercial supplier | Not applicable | Y-HPLC |
| Liu 2013 | Monacolin K, 2.5 mg  Monacolin complex | Commercial supplier | Not applicable | Y-HPLC |
| Jiang 2014 | Monacolin K, 2.5 mg  Monacolin complex | Commercial supplier | Not applicable | Y-HPLC |
| Yang 2014 | Monacolin K, 5.0 mg  Monacolin complex | Commercial supplier | Not applicable | Y-HPLC |
| Liu 2014 | Monacolin K, 2.5 mg  Monacolin complex | Commercial supplier | Not applicable | Y-HPLC |
| Tan 2015 | Monacolin K, 5.0 mg  Monacolin complex | Commercial supplier | Not applicable | Y-HPLC |
| Zhang 2015 | Monacolin K, 2.5 mg  Monacolin complex | Commercial supplier | Not applicable | Y-HPLC |
| Cui 2016 | Monacolin K, 2.5 mg  Monacolin complex | Commercial supplier | Not applicable | Y-HPLC |
| Pan 2016 | Monacolin K, 2.5 mg  Monacolin complex | Commercial supplier | Not applicable | Y-HPLC |
| Su 2016 | Monacolin K, 2.5 mg  Monacolin complex | Commercial supplier | Not applicable | Y-HPLC |
| Yang 2018 | Monacolin K, 5.0 mg  Monacolin complex | Commercial supplier | Not applicable | Y-HPLC |
| Jin 2019 | Monacolin K, 2.5 mg  Monacolin complex | Commercial supplier | Not applicable | Y-HPLC |
| Peng 2020 | Monacolin K, 5.0 mg  Monacolin complex | Commercial supplier | Not applicable | Y-HPLC |
| Li 2020 | Monacolin K, 5.0 mg  Monacolin complex | Commercial supplier | Not applicable | Y-HPLC |
| Xia 2021 | Monacolin K, 5.0 mg  Monacolin complex | Commercial supplier | Not applicable | Y-HPLC |
| Ma 2021 | Monacolin K, 5.0 mg  Monacolin complex | Commercial supplier | Not applicable | Y-HPLC |
| Zhang 2021 | Monacolin K, 2.5 mg  Monacolin complex | Commercial supplier | Not applicable | Y-HPLC |
